# Hernie obturatrice étranglée: à propos de deux cas

**DOI:** 10.11604/pamj.2015.20.169.3110

**Published:** 2015-02-24

**Authors:** Aggouri Younes, Benhammane Hossam, Yazough Issam, Toughrai Imane, Ait Laalim Said, Ibn Majdoub Karim, Mazaz Khalid

**Affiliations:** 1 Service de Chirurgie B, CHU Hassan II, Fès, Maroc

**Keywords:** Hernie obturatrice, occlusions, intestin grêle, obturator hernia, occlusions, small intestine

## Abstract

La hernie obturatrice (HO) est rare. Elle est à l'origine de 0,2 à 1,6% des occlusions mécaniques de l'intestin grêle avec un taux de mortalité et morbidité après chirurgie est respectivement de 35 et 18%. Nous rapportons le cas de deux patientes chez qui le diagnostic de HO étranglée est établie dans le cadre du bilan d'une occlusion. La HO est une entité dont le diagnostic préopératoire est difficile en raison de la faible spécificité clinique. L'examen tomodensitométrique semble être une aide majeure au diagnostic étiologique. Mais une fois le diagnostic d'occlusion posé, une intervention en urgence permettra d'en préciser l’étiologie et d'en réaliser le traitement. Tout retard thérapeutique majore la mortalité et la morbidité.

## Introduction

Une hernie obturatrice se définit comme l'issue d'une partie du contenu abdominal par le canal obturateur [1, 2]. Il s'agit d'une pathologie rare. Elles représentent 0,05 à 1,4% de toutes les hernies opérées et 0,2 à 1,6% des occlusions [2]. Le but de ce travail était d'envisager les différents aspects cliniques, thérapeutiques et diagnostiques de cette variété rare de hernie.

## Patient et observation

### Cas 1

**Figure 1:**
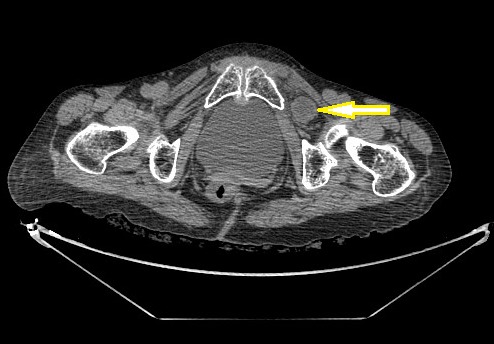
Tomodensitométrie abdominale, coupe axiale montrant une hernie obturatrice gauche

**Figure 2:**
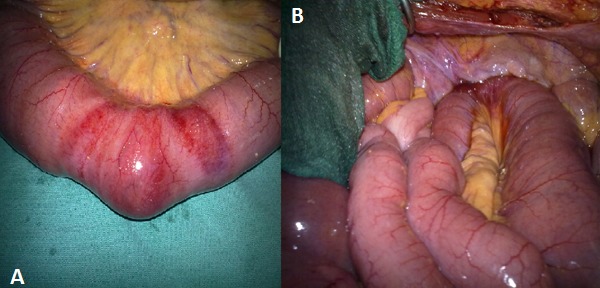
(A) pincement latéral de l'anse incarcéré; (B) anse incarcéré dans le trou obturateur avec disparité de caliber

Patiente de 60 ans mère de 6 enfants hypertendu jamais opérée mince et amaigrie qui présente un syndrome sub-occlusif évoluant 48 h avant son admission associé à quelque épisodes de vomissement alimentaire. A l'examen patiente apyrétique abdomen légèrement distendu tympanique a la percussion orifice herniaire libre, ampoule rectale vide. ASP: NHA de type grêlique. TDM: objective une HO gauche étranglée (Figure 1). Patiente opérée par laparotomie, l'exploration objective la présence d'une hernie obturatrice étranglée à gauche avec un pincement latéral responsable de la disparité de calibre (Figure 2 A, B), on a réalisé une réduction douce du grêle incarcéré qui été viable et fermeture du trou obturateur par plicature du péritoine pariétale, suite post opératoire simple.

### Cas 2

**Figure 3:**
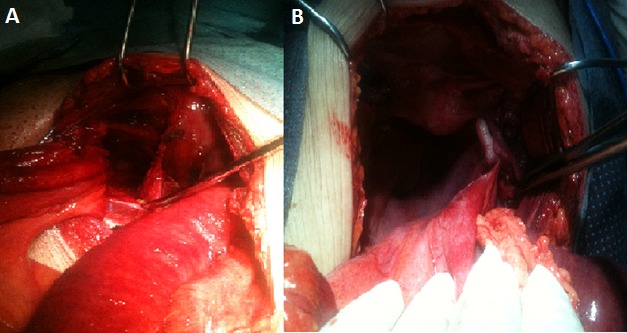
(A) trou obturateur; (B) plicature du péritoine pariétale et interposition de la trompe droite

Patiente de 75 ans mère de 5 enfants diabétique sous ado, cardiopathie hypertensive, jamais opérée qui présente un syndrome sub-occlusif évoluant 3 jours avant son admission associé à des vomissements alimentaire, l'examen clinique trouve une patiente apyrétique, abdomen légèrement distendu tympanique à la percussion avec un abdominale souple à la palpation et une hernie crurale droite étranglée. ASP: niveau hydro-aérique de type grêlique. Patiente opérée le même jour pour occlusion secondaire à une hernie crurale étranglée par voie élective ayant bénéficié d'une cure de la hernie selon lytle sans résection. Devant la non amélioration de la symptomatologie clinique et l'apparition de signes de péritonisme la patiente a été reprise à J2 par laparotomie et l'exploration a mis en évidence la présence d'une HO droite étranglée avec une anse grêle incarcéré nécrosé, on a réalisé une résection anastomose manuelle termino-terminale et fermeture du trou obturateur par plicature du péritoine pariétale et interposition de la trompe droite (Figure 3 A, B). L’évolution a été défavorable avec décès de la patiente dans un tableau de défaillance multiviscérale.

## Discussion

La hernie obturatrice étranglée est une pathologie rare [3, 4]. Le premier cas fut observé en 1718 (Lemaire, Strasbourg). On assiste à l'augmentation de sa prévalence secondaire au vieillissement de la population [5]. La HO est six fois plus fréquente chez la femme que chez l'homme [5, 6]. Les facteurs les plus souvent associés à l'apparition d'une HO étranglée on note l’âge avancé (à partir de 70 ans), le sexe féminin, la perte pondérale et la survenue d'une laxité du plancher pelvien liée à la multiparité [6]. La hernie obturatrice est généralement latente jusqu’à son étranglement et se révèle par un syndrome occlusif aigu, parfois précédé d’épisodes d’étranglement spontanément réduit dans 23,5% [6]. On retrouve dans la littérature un taux d’épisodes sub-occlusifs variant de 11,8 à 34, 7% [4, 6], ce qui a été noté chez nos deux patientes. La HO siège le plus souvent du côté droit, associée à une hernie inguinale dans 2,1% des cas [7], et elle est bilatérale dans 6% des cas. Dans les cas que nous avons rapportés, l'association à une hernie crurale chez la deuxième patiente a fait errer le diagnostic et aboutis à un retard du traitement chirurgical adéquat, qui a probablement été à l'origine du décès.

Le meilleur argument clinique est le signe de Romberg-Howship. Sa fréquence varie entre 15 et 50% des cas [3, 7]. Il correspond à une douleur liée à la compression du nerf obturateur, par le sac herniaire, notamment de sa branche cutanée. Elle est amplifiée par l'abduction et la rotation interne du pied il est connu comme étant pathognomonique de la hernie obturatrice. Il faut préciser que ce signe n'a pas été recherché chez nos deux patientes. En effet, compte tenu de la rareté de cette pathologie, et qu'il ne fait pas partie des signes cliniques recherchés systématiquement, lors de l'examen d'un patient ayant une occlusion aiguë de l'intestin grêle. Différents examens ont été utilisés pour le diagnostic de la HO. À l'heure actuelle, la TDM est l'examen de choix [6, 7]. L'absence de cause évidente à une occlusion intestinale aiguë du grêle ne doit pas conduire à une attitude conservatrice, mais à la réalisation précoce d'une TDM abdominale surtout chez les patientes âgées avec les facteurs de risques déjà décrit. Cet examen est actuellement le moyen le plus fiable pour établir le diagnostic de HO étranglée nécessitant une certaine expérience de la part des radiologues. Cet examen permet de raccourcir la période diagnostique, avant l'installation d'une nécrose intestinale, voire d'une péritonite, responsable d'une mortalité et d'une morbidité élevée [7].

Le traitement de la hernie obturatrice étranglée est chirurgical. Différentes approches sont envisageables, variables tant par leur voie d'abord, que par la technique de réparation. La laparotomie en urgence est l'approche la plus rapide et la plus sûre elle facilite la résection intestinale lorsque l'occlusion est compliquée de nécrose intestinale et l'exploration redresse le diagnostic [2, 3, 7]. Si le diagnostic est posé en préopératoire, la voie prépéritonéale est la plus appropriée, permettant un accès bilatéral aux régions fémorales, inguinale et obturatrice. Dans le cadre des occlusions du grêle, outre son rôle thérapeutique, la laparoscopie est un outil diagnostique, permettant de préciser le caractère organique et l’étiologie de l'occlusion [7]. Actuellement, même si cette technique a été rapportée, l'expérience pour le traitement des hernies obturatrice est encore trop limitée pour être recommandée comme procédure de routine. Le traitement chirurgical comporte une réduction douce et sans traction de l'anse congestive et fragile qui est incarcérée. Si après réduction, l'intestin est nécrosé, une résection économique s'impose. La réparation du défet peut être réalisée par simple suture ou par mise en place d'un matériel prothétique [7, 8]. Sans réparation chirurgicale, le taux de récidive est de 10% [8]. La réparation à l'aide de structures adjacentes (Exp: vessie) semble permettre une réparation plus stable que la fermeture péritonéale seule [8]. Classiquement, les réparations optimales utilisent des prothèses, non recommandées en cas de péritonite ou de perforation intestinale.

## Conclusion

Les hernies obturatrices sont une cause rare d'occlusion digestive dont le diagnostic préopératoire est difficile en raison de la faible spécificité clinique. L'examen tomodensitométrique semble être une aide majeure au diagnostic étiologique. Mais une fois le diagnostic d'occlusion posé, une intervention en urgence permettra d'en préciser l’étiologie et d'en réaliser le traitement. Tout retard thérapeutique majore la mortalité et la morbidité.
